# Knockdown of SETDB1 inhibits breast cancer progression by miR-381-3p-related regulation

**DOI:** 10.1186/s40659-018-0189-0

**Published:** 2018-10-11

**Authors:** Milu Wu, Baohua Fan, Qijing Guo, Yan Li, Rong Chen, Nannan Lv, Yinzhuo Diao, Yushuang Luo

**Affiliations:** Department of Oncology, The Affiliated Hospital of Qinghai University, No. 29, Tongren Rd, Xining, 810001 Qinghai China

**Keywords:** SET domain bifurcated 1, miR-381-3p, Breast cancer, Proliferation, Cell cycle progression, Migration

## Abstract

**Background:**

SET domain bifurcated 1 (SETDB1) has been widely considered as an oncogene playing a critical role in many human cancers, including breast cancer. Nevertheless, the molecular mechanism by which SETDB1 regulates breast cancer tumorigenesis is still unknown.

**Methods:**

qRT-PCR assay or western blot analysis was performed to assess the expression level of SETDB1 mRNA or protein, respectively. siSETDB1, pCMV6-XL5-SETDB1, miR-381-3p mimic, or miR-381-3p inhibitor was transfected into cells to regulate the expression of SETDB1 or miR-381-3p. MiRNA directly interacted with SETDB1 was verified by luciferase reporter assay and RNA immunoprecipitation. CCK-8 assay, colony formation assay, flow cytometric analysis, and transwell assay were used to detect the abilities of cell proliferation, cell cycle progression and migration, respectively. Animal model of xenograft tumor was used to observe the regulatory effect of SETDB1 on tumor growth in vivo.

**Results:**

We verified that SETDB1 mRNA level was upregulated in breast cancer tissues and cell lines, and SETDB1 depletion led to a suppression of cell proliferation, cell cycle progression and migration in vitro, as well as tumor growth in vivo. SETDB1 was verified to be a target of miR-381-3p. Moreover, miR-381-3p overexpression suppressed cell proliferation, cell cycle progression and migration, whereas SETDB1 abated miR-381-3p-mediated regulatory function on breast cancer cells.

**Conclusions:**

This study revealed that SETDB1 knockdown might suppress breast cancer progression at least partly by miR-381-3p-related regulation, providing a novel prospect in breast cancer therapy.

## Background

Breast cancer, one of the most common malignancy in women, is a leading cause of cancer death all over the world [1]. Although the developments of diagnosis and therapy techniques have highly improved the survival rate of breast cancer patients, up to 30% of patients died from palindromia and metastasis after having the standard-of-care therapy [2]. Therefore, it is of importance to explore the molecular mechanism of breast cancer pathogenesis for the effective therapy.

SET domain bifurcated 1 (SETDB1), also known as KMET1 or ESET, is a novel SET domain protein with histone H3 lysine 9 (H3K9)-specific methyltransferase activity which plays a pivotal role in early embryonic development [3]. It is recruited to the chromatin for silence promoters via the methyl-CpG-binding protein MBD1 [4] or some tumor inhibitor genes such as *P53BP2* and *RASSF1A* [5]. Accumulating evidences suggest that SETDB1 might function as a novel oncogene to be involved in multiple human cancers, such as hepatocellular carcinoma [6], lung cancer [7], and sporadic cutaneous melanoma [8]. Recently, a research document demonstrated that SETDB1 was regulated by miR-7 played a critical role in the metastasis of breast cancer [9]. Moreover, the abnormal expression of SETDB1 protein was found in human breast cancer cell lines by SILAC-based proteomic analysis [10]. Nevertheless, the molecular mechanism by which SETDB1 regulates breast cancer tumorigenesis is still unknown.

MicroRNAs (miRNAs), a type of short non-protein-coding RNAs with 20–22 nucleotides, are negative regulators of gene expression by base-pairing to the 3′ untranslated regions (3′-UTR) of mRNAs [11]. Following binding to partially complementary sites, miRNAs lead to a repression of translation and degradation of transcript [12]. Growing amount of evidences have suggested that miRNAs implicate in multiple physiological and developmental cellular processes, such as cell growth, differentiation, autophagy and apoptosis [13]. Dysregulation of some miRNAs has been widely acknowledged to be involved in a multitude of human cancers, including breast cancer [14]. Of these miRNAs, miR-126 was found to inhibit breast tumor proliferation and growth, and miR-335 repressed tumor metastasis [15]. Whereas, upregulated miR-10b was positively correlated with cell migration and invasion through targeting homeobox D10 (HOXD10) in breast cancer [16].

Here, we asked whether SETDB1 played a certain role partly by miRNAs regulation in breast cancer. We found that SETDB1 level was upregulated in breast cancer, and SETDB1 knockdown repressed tumor growth in vitro and in vivo. Moreover, SETDB1 was verified to be a functional target of miR-381-3p. Therefore, this study hinted that SETDB1 knockdown might repress breast cancer progression at least partly by miR-381-3p-related regulation, highlighting a novel therapeutic target for breast cancer treatment.

## Methods

### Clinical tissues collection

Forty five pairs of breast cancer tissues and adjacent normal breast tissues were obtained from patients who underwent radical mammectomy at the Affiliated Hospital of Qinghai University. No systemic or local treatment was performed in these patients before surgery. Tissue samples were immersed in RNAlater (Qiagen, Hilden, Germany) and snap frozen immediately, followed by stored at − 80 °C until used. Written informed consent were obtained from all patients prior to the study, and the study was approved by the Institutional Review Broad and Ethical Committee of Affiliated Hospital of Qinghai University.

### Cell culture

Human mammary epithelial cell line (MCF-10A) and breast cancer cell lines (MCF-7, MDA-MB-231) that were purchased from American Type Culture Collection (ATCC, Manassas, VA, USA), were maintained in Dulbecco’s Modified Eagle Medium (DMEM, Gibco, Rockville, MD, USA) with 10% fetal bovine serum (FBS, Gibco), 1% penicillin/streptomycin (Gibco) in a humidified incubator (5% CO_2_) at 37 °C.

### Cell transfection

Small interference RNA targeting SETDB1 (siSETDB1) and the homologous negative control (siNC) were purchased from Applied Biosystems (Foster city, CA, USA). Modified miRNA mimic for hsa-miR-381-3p (miR-381-3p mimic) and its cognate negative control (miR-NC), miR-381-3p inhibitor and its negative control (inhibitor-NC), SETDB1 overexpression plasmid (pCMV6-XL5-SETDB1) were commercially synthesized by GenePhama (Shanghai, China). As previously described [17], 10 nM of oligonucleotides or 2 µg of plasmids were transfected into breast cancer cells using Lipofectamine^®^ RNAiMAX™ transfection reagent (Invitrogen, Waltham, MA, USA) referring to the manual of application.

### Quantitative real-time PCR (qRT-PCR)

Total RNA was extracted from tissues and cells using TRIzol™ plus RNA Purification Kit (Invitrogen) according to the manual of application. RNA quality was evaluated by an Agilent 2100 Bioanalyzer (Agilent Technologies, Santa Clara, CA, USA). Then, a total of 500 ng RNA was reverse-transcribed into cDNA using the M-MLV reverse transcriptase (Promega, Madison, WI, USA). qRT-PCR analysis was performed in triplicates with the SsoFast™ EvaGreen Supermix (Bio-Rad, Hercules, CA, USA) on a StepOnePlus™ Real-Time PCR System (Applied Biosystems). The expression level of SETDB1 mRNA was calculated using 2^−ΔΔCt^ algorithm and normalized to that of GAPDH. The following primers were used: SETDB1 mRNA: 5′-GACTCTCTGAGACAACTTCCAAGGA-3′ (forward) and 5′-CAGGGATTGAGGGAGGAACA-3′ (reverse); GAPDH: 5′-TGCACCACCAACTGCTTAGC-3′ (forward) and 5′-GGCATGCACTGTGGTCATGAG-3′ (reverse).

### Cell proliferation assay

Cell proliferation capacity was detected by Cell Counting Kit-8 Detection Kit (CCK-8, Dojindo Molecular Technologies, Shanghai, China) referring to the protocol of manufacturer. In brief, at 0, 24, 48, and 72 h after transfection, 10 µl of CCK-8 solution was added to each well and incubated at 37 °C for 3 h. Absorbance value at 450 nm was measured by a microplate reader (Bio-Rad).

### Colony formation assay

Transfected cells were trypsinized into a single-cell suspension and were cultured in growth medium at 37 °C for 14 days to form natural colony. Then, the colonies were fixed with 4% paraformaldehyde (Sigma-Aldrich, St. Louis, MO, USA) and stained with 0.5% crystal violet (Sigma-Aldrich).

### Flow cytometric analysis of cell cycle progression

Cell cycle progression was assessed by flow cytometry with Cell Cycle and Apoptosis Analysis Kit (Beyotime Institution of Biotechnology, Shanghai, China). Briefly, at 48 h after transfection, cells were harvested and fixed in pre-cooling 70% ethanol overnight. Following PBS-washing three times, the cells were stained with 100 µg/ml propidium iodide (PI) at 37 °C for 30 min. The evaluation of cell cycle was performed using the Modfit 3.0 software (Verity Software House, Topsham, ME, USA) on a FACScalibur flow cytometer (Becton–Dickinson, Franklin Lakes, NJ, USA).

### Cell migration assay

Transfected cells were seeded on the upper chamber of an 8-µm pore size Transwell (Corning, Toledo, NY, USA) to detect the cell migration ability. Upper medium was replaced by serum-free medium, while the lower chamber contained growth medium with 10% FBS. After incubation for 24 h, migrated cells were fixed in methanol and stained with 0.5% crystal violet (Sigma-Aldrich). At last, the number of migrated cells was counter with a microscope (Leica, Wetzlar, Germany).

### Luciferase reporter assay

A fragment of SETDB1 3′-UTR containing the putative binding site of miR-381-3p, was amplified from human genomic DNA and inserted into a modified pGL3 Luciferase Reporter Vector (Promega) to construct wild SETDB1 3′-UTR report vector (SETDB1 3′-UTR-WT). To construct mutated SETDB1 3′-UTR report vector (SETDB1 3′-UTR-MUT), the residues that base-pairs with miR-381-3p were mutated by site-directed mutagenesis with Q5 Site-Directed Mutagenesis Kit (New England Biolabs, Ipswich, MA, USA). 20 µg of SETDB1 3′-UTR-WT or SETDB1 3′-UTR-MUT construct was cotransfected with 200 ng of miR-381-3p mimic or miR-381-3p inhibitor into MDA-MB-231 or MCF-7 cells. 48 h later, the luciferase activity was measured using a Lumat LB9508 luminometer (Berthold, Bad Wildbad, Germany).

### RNA immunoprecipitation (RIP)

Coimmunoprecipitation (co-IP) experiment of miRNA with anti-Argonaute1 (anti-Ago1, Abcam, Cambridge, UK) was performed as previously reported [18]. Briefly, cells were transfected with miR-NC or miR-381-3p mimic for 48 h, and were lysed with cell lysis buffer (25 mM Tris–HCl, pH = 7.5, 150 mM KCl, 2 mM EDTA, 0.5% NP-40, 1 mM DTT, 100 U/ml RNasin). Then, the complex of anti-Ago1 and Protein A magnetic beads was added to cell lysates, and incubated at 4 °C overnight to get the immunoprecipitation complex. Lastly, the enrichment of SETDB1 mRNA was measured by qRT-PCR assay and anti-IgG (Abcam) was as negative control.

### Western blot analysis

Protein fractions were obtained from cells using ice-cold RIPA buffer (50 mM Tris–HCl, pH = 7.5, 150 mM NaCl, 1% TritonX-100, 1 mM EDTA, 2.5 mM sodium pyrophosphate, 1 mM β-glycerophosphate) supplemented with protease and phosphatase inhibitor cocktails (Roche Diagnostics, Mannheim, Germany). About 50 µg of protein extractive was subjected to 10% sodium dodecyl sulfate polyacrylamide gel electrophoresis gel (10% SDS-PAGE) and transferred to polyvinyldene fluoride (PVDF) membrance (Bio-Rad). Followed by blocking with 5% non-fat milk at room temperature for 1 h, the membrances were incubated with SETDB1 (1:2000, Abcam) and β-actin (1:5000, Abcam) antibodies at 4 °C overnight. Then, the membrances were further probed with horseradish peroxidase-conjugated secondary antibodies (1:5000, Cell Signaing Technology, Danvers, MA, USA) for 1 h at room temperature. All protein bands were analyzed using an Image-Pro plus 4.5 software (Media Cybernetics, Silver Spring, MD, USA).

### In vivo animal model

All animals used were under an approved protocol of the Institutional Animal Care and Use Committee of Affiliated Hospital of Qinghai University. 6–8 weeks male BALB/c mice were purchased from Qinghai Research Center of Laboratory Animal (Xining, China) and were housed in a specific-pathogen-free environment. For xenograft tumors formation, 1.0 × 10^6^ MCF-7 cells transfected with siNC or siSETDB1 were subcutaneously injected into the nude mice (n = 8 per group). Tumor volume was determined with a caliper every 10 days. On 50 days after cell implantation, mice were euthanized and tumors were excised for weight assessment and qRT-PCR assay of SETDB1 expression.

### Statistical analysis

All data were presented as mean ± standard deviation (SD) from three independent experiments. Differences between two groups were compared using Student’s *t*-test. Kaplan–Meier method was used to evaluate overall survival, and log-rank test was performed to analyze the difference in survival between two groups. *P* value of < 0.05 was considered statistically significant.

## Results

### Upregulation of SETDB1 mRNA in breast cancer tissues and cell lines

**Fig. 1:**
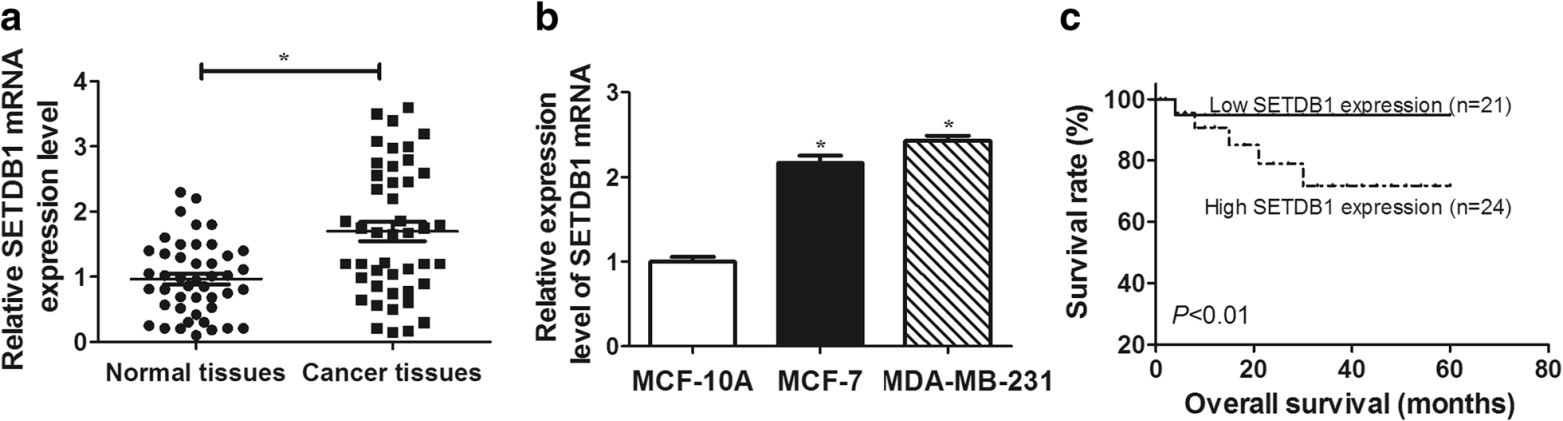
SETDB1 mRNA expression was upregulated in breast cancer tissues and cell lines. **a** SETDB1 mRNA expression was measured in 45 pairs breast cancer tissues and adjacent normal breast tissues by qRT-PCR assay. **b** qRT-PCR assay of SETDB1 mRNA level in breast cancer cell lines (MCF-7, MDA-MB-231) and human mammary epithelial cell line (MCF-10A). **c** Kaplan–Meier survival assay and log-rank test were used to evaluate the correlation between SETDB1 mRNA level and breast cancer patient prognosis in 45 breast cancer patients in low- and high-risk groups based on SETDB1 mRNA expression. *P < 0.05 vs. corresponding control

The expression level of SETDB1 mRNA in breast cancer tissues and cell lines was firstly assessed by qRT-PCR assay. As shown in Fig. 1a, the relative expression of SETDB1 mRNA was significantly elevated in breast cancer tissues compared with normal tissues. The data also revealed that SETDB1 mRNA level was higher in breast cancer cell lines than that in normal MCF-10A cells (Fig. 1b).

Subsequently, Kaplan–Meier survival assay and log-rank test using patient post-operative survival were performed to further determine the correlation between the expression level of SETDB1 mRNA and the prognosis of breast cancer patients. According to the median ratio of SETDB1 mRNA expression, the 45 breast cancer patients were classified into two groups: high SETDB1 mRNA expression group (n = 24) and low SETDB1 mRNA expression group (n = 21). Kaplan–Meier survival curve presented that low SETDB1 mRNA expression group had markedly longer survival times compared to those with high SETDB1 mRNA expression group (*P* < 0.01, Fig. 1c). Taken together, these data hinted that abnormal expression of SETDB1 might be associated with the progression of breast cancer.

### SETDB1 knockdown inhibited proliferation, cell cycle progression and migration in breast cancer cells in vitro

**Fig. 2:**
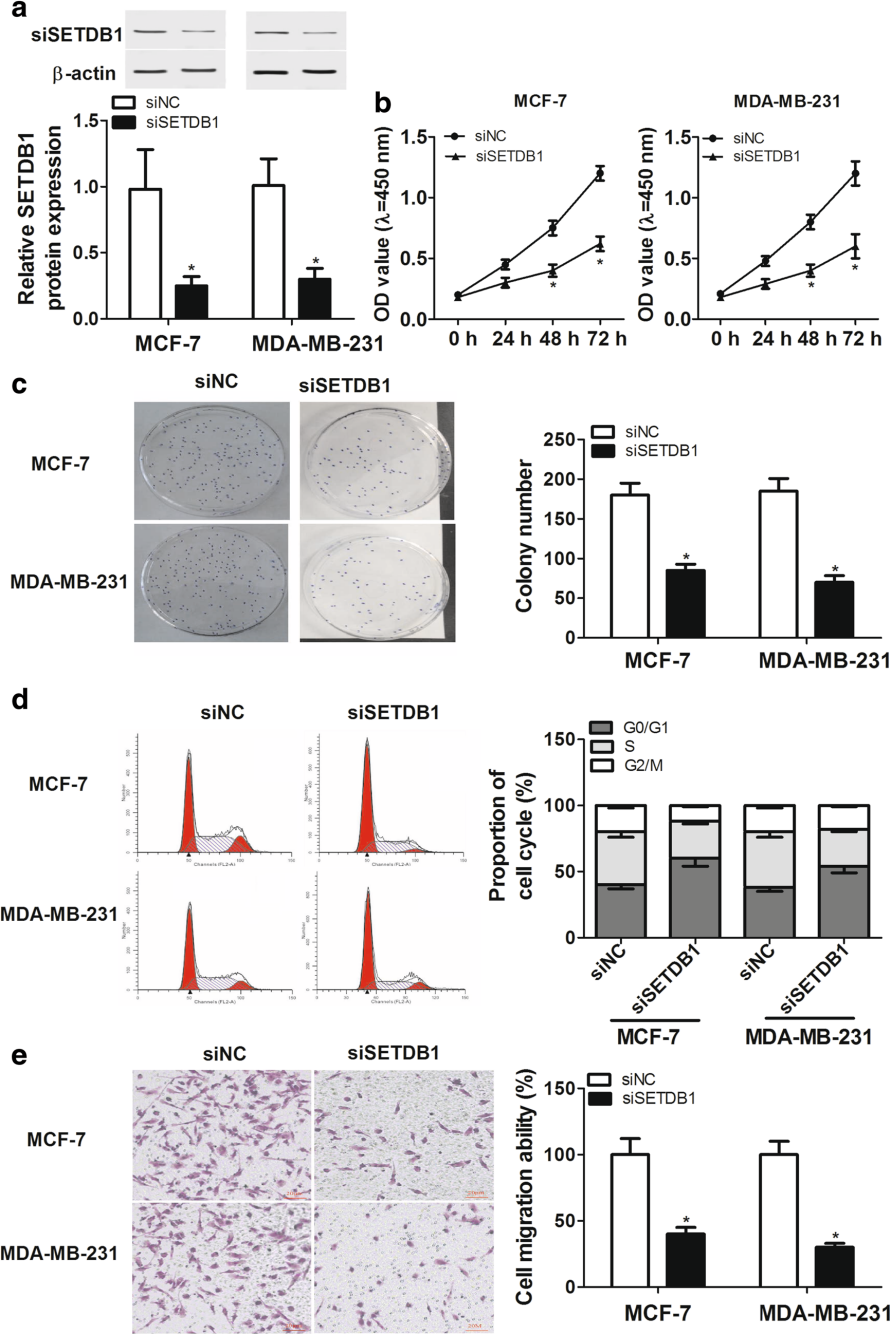
SETDB1 knockdown suppressed proliferation, cell cycle progression and migration in MCF-7 and MDA-MB-231 cells. Western blot assay of SETDB1 expression (**a**), CCK-8 assay (**b**) and colony formation assay (**c**) of cell proliferation capacity, flow cytometry analysis of cell cycle progression (**d**), and transwell assay of cell migration ability (**e**) in MCF-7 and MDA-MB-231 cells transfected with siNC or siSETDB1. *P < 0.05 vs. siNC

To explore the function of SETDB1 on breast cancer progression, loss-of-function analyses were performed by silencing the gene expression with siRNA targeting SETDB1 (siSETDB1) in vitro. Western blot assay displayed that SETDB1 protein level was drastically repressed by transfection with siSETDB1 compared with that of control in MCF-7 and MDA-MB-231 cells (Fig. 2a). Interestingly, CCK-8 and colony formation assays showed that MCF-7 and MDA-MB-231 cells in the siSETDB1 groups had lower proliferation capacity and colony formation efficiency compared to homologous control (Fig. 2b, c). Moreover, SETDB1 depletion hampered cell cycle progression through triggering the G0/G1 accumulation in MCF-7 and MDA-MB-231 cells (Fig. 2d). In cell migration assays, exogenous downregulation of SETDB1 expression remarkably repressed cell migration ability (Fig. 2e). All these results suggested that SETDB1 knockdown might be sufficient to inhibit cell proliferation, cell cycle progression and migration in breast cancer cell lines in vitro.

### Knockdown of SETDB1 repressed tumor growth in vivo

**Fig. 3:**
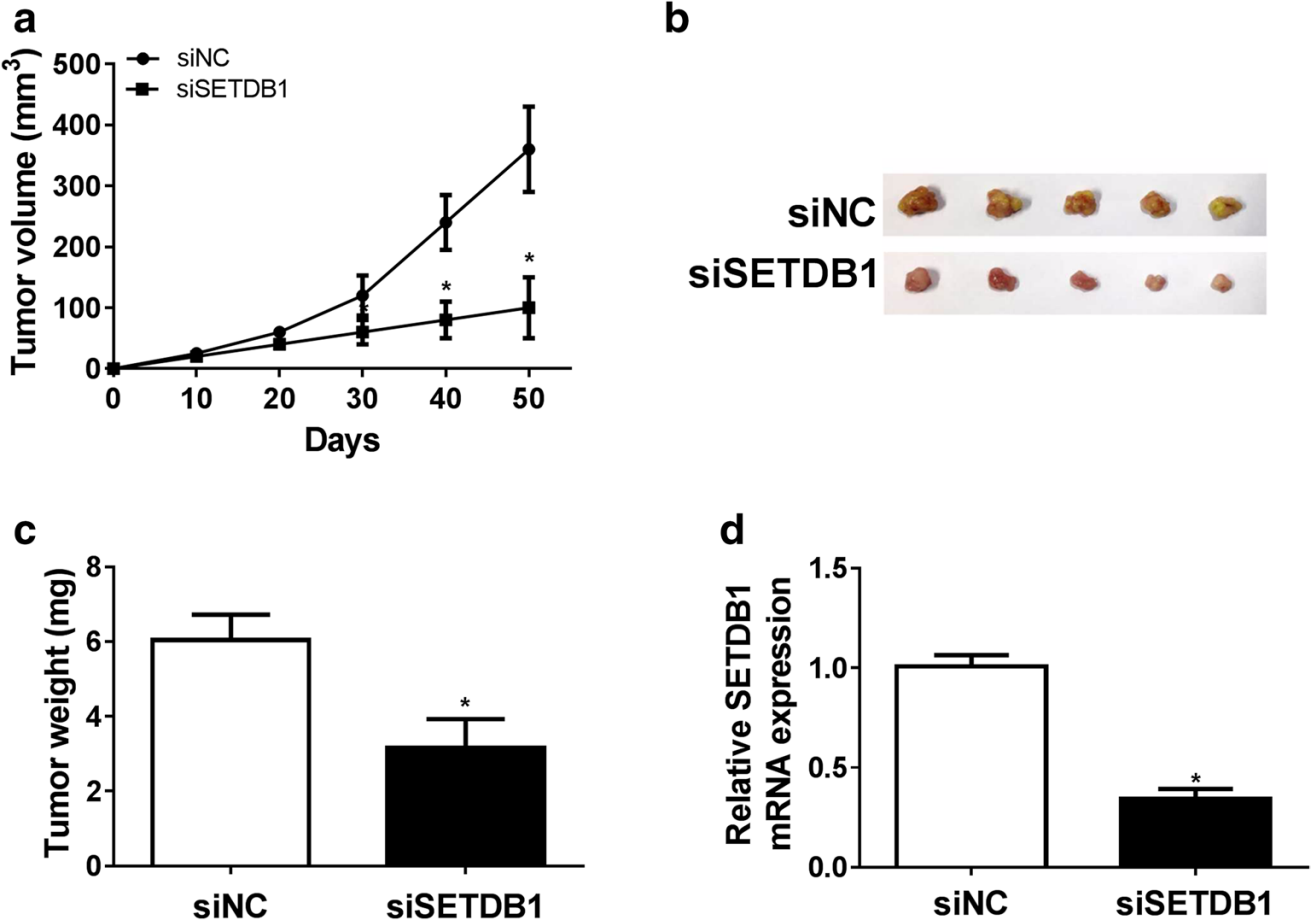
SETDB1 depletion repressed tumor growth in vivo. Nude mice were subcutaneously injected with 1.0 × 10^6^ MCF-7 cells transfected with siNC or siSETDB1. 50 days later, mice were killed and tumor masses were removed. **a** Tumor volume was mearsured by a caliper every 10 days. **b** Bright-field imaging of the xenograft tumors. **c** The average weight of the xenograft tumors. **d** qRT-PCR assay of SETDB1 mRNA expression in removed tumor tissues. *P < 0.05 vs. siNC control

Then, MCF-7 cells transfected with siSETDB1 were subcutaneously inoculated into the mice to evaluate whether SETDB1 could regulate tumor growth in vivo. These results revealed that SETDB1 knockdown highly repressed tumor growth, presented as the decrease of tumor volume (Fig. 3a) and tumor weight (Fig. 3b, c). Moreover, as shown in Fig. 3d, SETDB1 mRNA level was significantly downregulated in tumors derived from siSETDB1-transfected cells compared to that of control. These data implied that SETDB1 knockdown might suppress tumorigenesis in vivo.

### Regulation of SETDB1 by miR-381-3p in a direct interaction

**Fig. 4:**
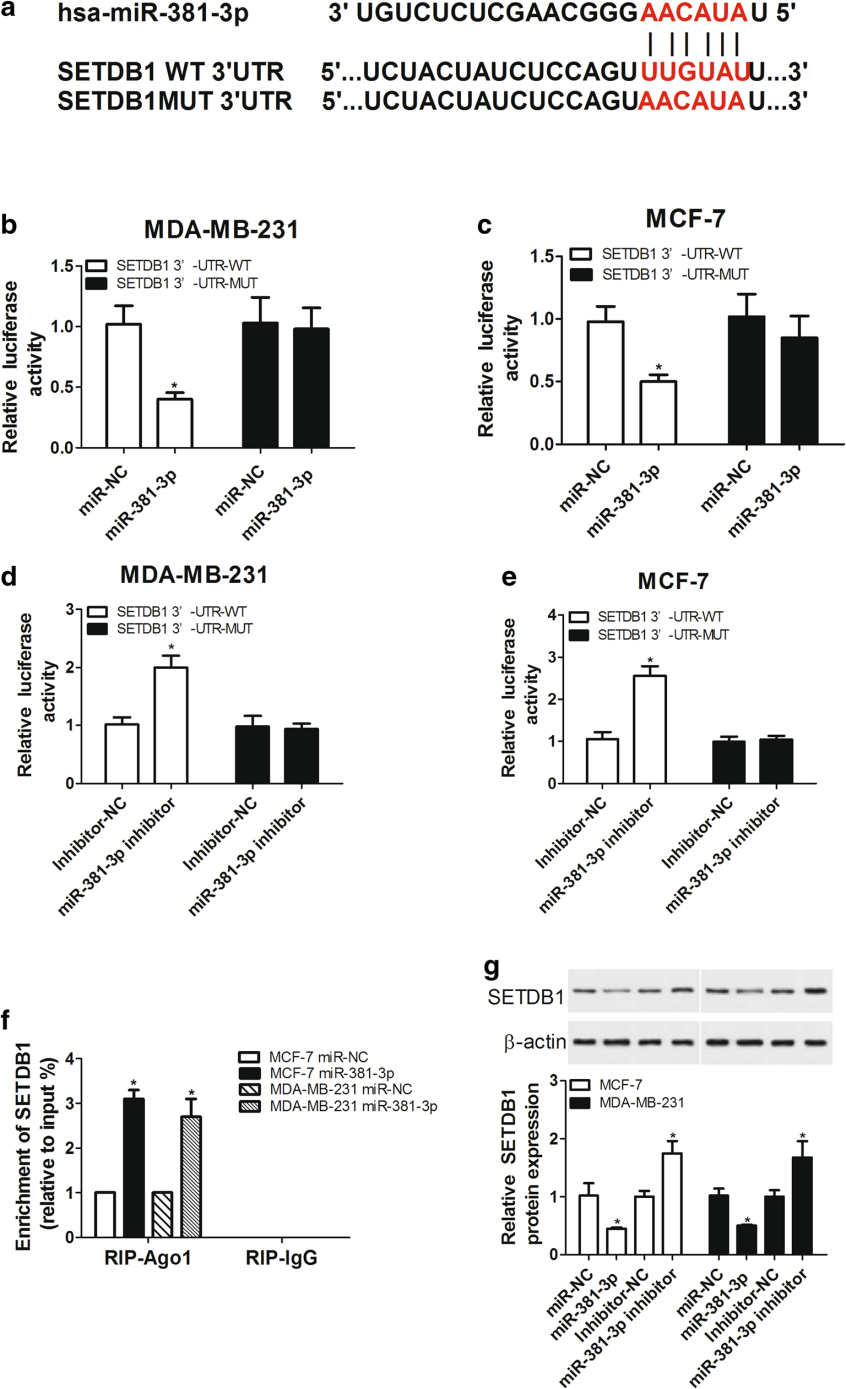
SETDB1 was regulated by miR-381-3p in a direct interaction in breast cancer cell lines. **a** Schematic of potential binding sites for miR-381-3p in the SETDB1 3′-UTR, the seed and the mutated sequences of potential binding sites. Luciferase reporter assays were performed to verify the interaction between miR-381-3p and SETDB1 by cotransfection with SETDB1 3′-UTR-WT or SETDB1 3′-UTR-MUT construct and miR-381-3p mimic into MDA-MB-231 cells (**b**) and MCF-7 cells (**c**), or by cotransfection with SETDB1 3′-UTR-WT or SETDB1 3′-UTR-MUT construct and miR-381-3p inhibitor into MDA-MB-231 cells (**d**) and MCF-7 cells (**e**). **f** RIP analysis was used to evaluate the binding between miR-381-3p and SETDB1 using anti-Ago1 in MCF-7 and MDA-MB-231 cells transfected with miR-381-3p mimic, followed by measurement of SETDB1 mRNA by qRT-PCR assay. **g** Western blot analysis of SETDB1 protein expression in MCF-7 and MDA-MB-231 cells transfected with miR-381-3p mimic or miR-381-3p inhibitor. *P < 0.05 vs. respective negative control

To further understand the molecular mechanism by which SETDB1 regulated tumor growth, online database was used to search for the miRNAs directly interacted with SETDB1 in breast cancer. Among the predicted miRNAs, miR-381-3p was chose for further research because it was confirmed to be involved in the development and progression of breast cancer [19]. The SETDB1 mRNA contained a 3′-UTR segment that was partially complementary to miR-381-3p (Fig. 4a). Subsequently, luciferase reporter assay and RNA immunoprecipitation (RIP) assay were performed to verify the correction between SETDB1 and miR-381-3p in breast cancer cells. For luciferase reporter assay, the partially sequence of SETDB1 3′-UTR was inserted into pGL3 Luciferase Reporter Vector as described previously [20]. SETDB1 3′-UTR-WT or SETDB1 3′-UTR-MUT construct was cotransfected with miR-381-3p mimic or miR-381-3p inhibitor into MDA-MB-231 or MCF-7 cells. The data revealed that the luciferase activity of SETDB1 3′-UTR-WT construct was significantly reduced by miR-381-3p overexpression (Fig. 4b, c), while it was highly enhanced by miR-381-3p depletion (Fig. 4d, e) in MDA-MB-231 and MCF-7 cells. Whereas, little change was found in the luciferase activity of SETDB1 3′-UTR-MUT construct in response to the alteration of miR-381-3p expression (Fig. 4b–e). RIP analysis showed that SETDB1 mRNA was specifically recruited to the miRNP complexes isolated with anti-Ago1 following miR-381-3p transfection in MDA-MB-231 and MCF-7 cells (Fig. 4f). Additionally, we further detected whether SETDB1 expression was regulated by miR-381-3p in MDA-MB-231 and MCF-7 cells. Western blot analysis showed that compared with corresponding counterparts, SETDB1 expression was markedly weakened by transfection with miR-381-3p mimic, while it was remarkably promoted following miR-381-3p inhibitor introduction (Fig. 4g). All these results indicated that SETDB1 was a direct target of miR-381-3p.

### Restoration of SETDB1 expression abrogated the regulatory function of miR-381-3p in breast cancer cell lines

**Fig. 5:**
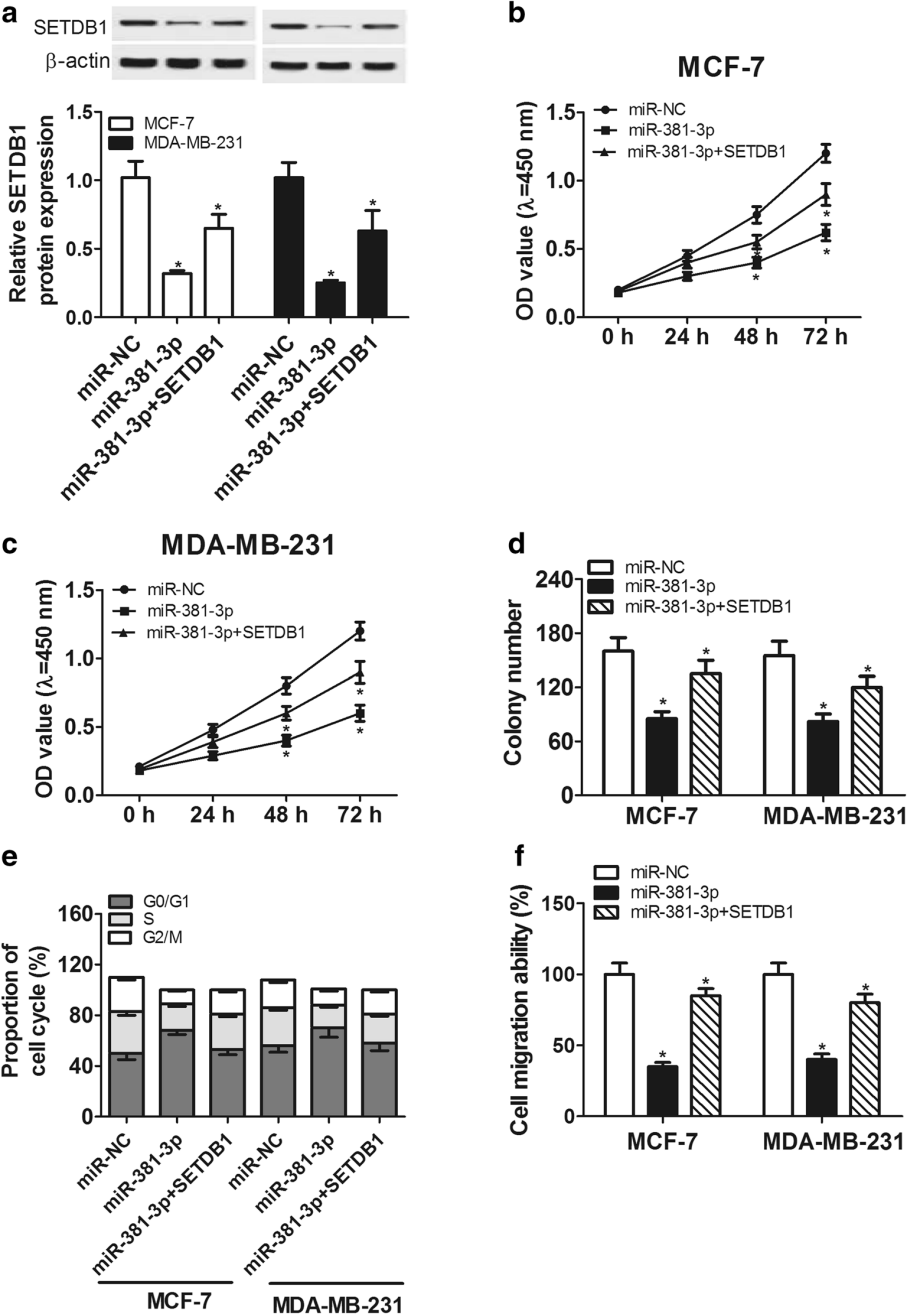
SETDB1 abrogated miR-381-3p-mediated inhibition on proliferation, cell cycle progression and migration in breast cancer cell lines. **a** Western blot analysis of SETDB1 expression, **b**, **c** CCK-8 assay and **d** colony formation assay of cell proliferation capacity, **e** flow cytometry analysis of cell cycle progression, and **f** transwell assay of cell migration ability in MCF-7 and MDA-MB-231 cells transfected with miR-381-3p mimic or miR-381-3p mimic + pCMV6-XL5-SETDB1. *P < 0.05 vs. corresponding control (miR-NC or miR-381-3p mimic)

Then, miR-381-3p mimic was transfected into MCF-7 and MDA-MB-231 cells to assess the function of miR-381-3p on breast cancer progression. As shown in Fig. 5a, SETDB1 expression was markedly inhibited by transfection with miR-381-3p mimic. Functional analysis revealed that miR-381-3p overexpression led to the suppression of cell proliferation capacity (Fig. 5b, c), colony formation ability (Fig. 5d), and cell cycle progression (Fig. 5e), as well as cell migration ability (Fig. 5f) in MCF-7 and MDA-MB-231 cells. Together, these data suggested that miR-381-3p might suppress cell proliferation, cell cycle progression and migration in breast cancer cell lines.

Further, to explore whether the suppression function of miR-381-3p was mediated by SETDB1, MCF-7 and MDA-MB-231 cells were cotransfected with miR-381-3p mimic and SETDB1 overexpression vector (pCMV6-XL5-SETDB1). The data illustrated that miR-381-3p-induced the suppression of SETDB1 expression was drastically abated by cotransfection with pCMV6-XL5-SETDB1 (Fig. 5a). Moreover, the restoration of SETDB1 expression remarkably abrogated miR-381-3p-mediated inhibition effect on cell proliferation capacity, colony formation ability, cell cycle progression and migration ability in MCF-7 and MDA-MB-231 cells (Fig. 5b–f). All these findings hinted that the restoration of SETDB1 expression undermined miR-381-3p-mediated retardation on proliferation, cell cycle progression and migration in breast cancer cell lines.

## Discussion

Histone lysine methylation has been reported to be implicated in transcriptional activation or suppression, which was of increasing interest owing to its involved in the neoplastic transformation [21]. H3K9 methylation was reported to play a vital role in multiple human cancer [22]. SETDB1, located at chromosome 1q21, is a member of H3K9 methylation catalyzed by histone lysine methyltransferases [23]. Over the past few years, SETDB1 has been widely considered as an oncogene playing a critical role in many human cancers. It was previously reported that SETDB1 protein level was upregulated in human melanomas tissues and it enhanced the formation of melanoma in a zebrafish model [24]. Silence of SETDB1 was found to inhibit lung tumor growth in vitro and in vivo, while it upregulation promoted the tumor invasiveness, highlighting its role as a novel therapeutic target [25]. SETDB1 also was reported to be overexpressed in hepatocellular carcinoma (HCC), which inactivation repressed the growth of HCC cell lines through regulating p53 methylation [26]. In addition to the cancers mentioned above, SETDB1 was proposed as an oncogene in prostate cancer [27], gliomas [28] and colorectal cancer [29]. In this study, SETDB1 level was verified to be elevated in breast cancer tissues and cell lines, which was consistent with the finding of Zhang et al. [9]. From a functional standpoint, SETDB1 knockdown inhibited breast tumor growth in vitro and in vivo. All these data suggested that SETDB1 knockdown might suppress breast cancer progression.

MiRNAs are attractive candidates as upstream regulators of tumor progression because they have been demonstrated to repress a set of target genes expression in a post-transcriptional way [30]. For instance, miR-135 and miR-203 inhibited breast tumor growth and metastasis in vitro and in vivo by targeting runx2 [31]. MiR-155 was found to accelerate tumor angiogenesis by directly suppressing von Hippel–Lindau (VHL) expression in triple-negative breast cancer [32]. It was reported that miR-621 overexpression promoted breast cancer chemosensitivity through targeting FBXO11 [33]. Moreover, miR-21 enhanced epithelial-to-mesenchymal transition (EMT) by inhibiting PTEN protein expression in breast cancer [34]. PTPRN2, MERTK, TNC and SOX4 were identified to be targets of miR-335 [15], AIB1 and CCND1 were the targets of miR-17-5p [35], and H-RAS and HMGA2 were verified as targets of let-7 [36] in breast cancer.

Then, software algorithms were performed to search for the miRNAs directly interacted with SETDB1 in breast cancer. Among the predicted miRNAs, miR-381-3p was chose for further research owing to its involvement in the progression of multiple human cancers. For example, in oral squamous cell carcinoma, miR-381-3p suppressed cell proliferation and cell cycle progression while enhanced apoptosis through directly targeting fibroblast growth factor receptor 2 (FGFR2) [37]. In non-small cell lung cancer, miR-381 led to a suppression of tumor growth and chemoresistance by direct downregulation of differentiation 1 (ID1) [38]. Additionally, a recent document reported that miR-381 suppressed proliferation, EMT and metastasis of breast cancer cells through targeting CXCR4 [19]. In the present study, SETDB1 was verified to be a functional target of miR-381-3p in breast cancer cells. Consistent with the findings of Xue et al. [19], miR-381-3p was manifested to suppress breast cancer cells proliferation, cell cycle progression and migration. Moreover, miR-381-3p-mediated regulatory function was abrogated by the restoration of SETDB1 expression.

## Conclusions

In conclusion, our study demonstrated that SETDB1 was upregulated in breast cancer and SETDB1 knockdown suppressed breast cancer progression at least partly by miR-381-3p-related regulation, highlighting SETDB1 as a novel biomarker for breast cancer therapy.

## Abbreviations

SETDB1: SET domain bifurcated 1
H3K9: H3 lysine 9
3′-UTR: 3′ untranslated regions
HOXD10: homeobox D10
siSETDB1: small interference RNA targeting SETDB1
qRT-PCR: quantitative real-time PCR
RIP: RNA immunoprecipitation
co-IP: coimmunoprecipitation
PVDF: polyvinyldene fluoride
